# Digital Dental Photography: A Contemporary Revolution

**DOI:** 10.5005/jp-journals-10005-1217

**Published:** 2013-10-14

**Authors:** Vela Desai, Dipika Bumb

**Affiliations:** Professor and Head, Department of Oral Medicine and Radiology, Jaipur Dental College, Jaipur, Rajasthan, India, e-mail: veladesai@hotmail.com; Department of Oral Medicine and Radioldiagnosis, Indian Cancer Society, New Delhi, India

**Keywords:** Digital photography, Photogrammetry, SLR cameras, Teledentistry

## Abstract

**Introduction:** Photographs are symbolic of memories and with the advent of digital photography it has become much easier to collect them in a second in a more comprehensive and qualitative manner. Technological advancements in the field of digital photography have revolutionized the concept of photography as a powerful medium of expression and communication. It also offers a spectrum of perception, interpretation and execution. Photography and dentistry go hand in hand for revelation of the hidden and overlooked defects in teeth and other parts of the cavity. This article emphasizes on the significance of digital photography in dentistry and guidelines for capturing orofacial structures and radiographs in a more accurate and informative manner.

**Conclusion:** Dental world constitutes of microstructures that have to be recorded in a detailed manner in order to perform patient education, documentation of records and treatment, illustration of lectures, publication and web connectivity of complicated cases.

**How to cite this article:** Desai V, Bumb D. Digital Dental Photography: A Contemporary Revolution. Int J Clin Pediatr Dent 2013;6(3):193-196.

## INTRODUCTION

Every photograph lacks expressional quality without a smile that is choreographed by dentists and due to the arrival of digitized systems it is easy to record miniature details about the pathology as well as the procedure that has to be carried out in a particular patient. Formulation of a proper diagnosis needs repeated insights in the data of patient, and this is made possible by the digital cameras.^1^ Digital cameras have numerous advantages over conventional photography like immediate and prompt accessibility to user via computers; instant errors can be spotted and retake in the same appointment, no illumination problem-no extra equipment, economic-no expenditure of films and its processing, can be easily transferred by electronic mails for consultation and are easy to store without worrying for fading of films, maintenance of study models and dental casts. Digital records have utmost importance to solve tedious forensic cases.^2^

Digital photography is described as the images that are stored in a computerized file format referred to as a digital image file. It signifies a file format that is composed of a graphical image instead of text or program data. These images can be recorded in the form of bitmapped image (JPEG, PNG, GIF, TIFF and BMP) and vector based images used in paint or illustration programs.^3^ DICOM stands for Digital Imaging and Communications in Medicine, is a standardized worldwide program that provides a common language for formatting and exchanging medical and dental data efficiently.

### Digital Photography Systems: The Past and the Future

In the 1960s, George Smith and Willard Boyle invented charge-coupled device (CCD) at bell labs followed by Kodak scientists (1986), invented the world's first megapixel sensor, capable of recording 1.4 million pixels producing 5 × 7-inch digital photo-quality print.^4^ In 1990, Logitech flooded the markets with digital consumer camera known as the Dycam Model 1 digicam. The Apple QuickTake 100 camera was the first digital camera that could be connected to the home computer via a serial cable in 1994. This camera featured a 640 × 480 pixel CCD which produced eight images stored in internal memory and a built-in flash.^5^

But now in this new era, digital cameras can be divided into compact and professional cameras. Their requirement is based on technical knowledge and field of use. Professional cameras (SLR – Single Lens Reflex) are generally more durable and provide better options to change the picture quality according to needs and surroundings. These cameras are comprised of the following important components like lens: a 35 mm zoom lens has ‘macro’ settings which allow focusing of close-up objects and organs and a true macrolens enables to focus down to even 1:1 magnification. They also standardize the Westminster scales. Dual point flash, ring flash: it has the ability to change the angle of the flash, reduce reflection and give shots in more depth and texture.^6^

Looking over these benefits, digital technology has changed the perspective of a dentist toward data collection, academics and treatment aspects. Intraoral cameras are available to capture the image of a tooth or different lesions in the oral cavity from different angles within seconds. Therefore, a full set of intra- and extraoral photos are best advised for every particular patient before and after the treatment along with videos of the procedures done.^7^ In digital photography a film is replaced by an electronic sensor that captures the image, i.e. a charged couple device (CCD) or complimentary metal oxide semiconductor (CMOS). CCDs are commonly used as they are more sensitive to light with better image quality than CMOS sensors, but they are more expensive to manufacture and use high power.^8^

A digital sensor is made up of millions of tiny photosensitive diodes called pixels which are coordinated in a matrix. Each pixel is a numerical value that corresponds to the 256 shades of gray at a single point in the image. Digital approximation of the image is done tracing the analog to digital conversion within the sensors through which a copy of the original image can be reconstructed. To record colors, a red, green or blue filter is placed over the individual sensor. It affects the resolution of camera and aids in larger print outs.^9^ Appropriate recommendation for an image is at least 24 bits of color depth and a resolution between 500 and 700 dpi.^10^

A new innovation has arrived in the field of dentistry called photogrammetry through which the geometric properties of objects can be determined from photographic images and has remarkably proved its usefulness for studying the three-dimensional occlusion of dental arches, teeth and their dimensions in orthodontics as well as prosthodontics.^11^

### Guidelines for Clinical Photography

To achieve a good digital dental photograph, standardization is very important, i.e. consistent lighting, exposure, patient positioning, perspective, depth of field and background. Photographs should be stored and presented appropriately for their use in publications.^12^ Three types of intraoral cameras are used like 35 mm film camera with macrolens and ring flash, intraoral video camera (Orthoscan camera) or 5 and 6 megapixel extraoral digital cameras.^13^ Several views should be taken for all the patients like frontal view that incorporates full facial profile and entire dentition. Other views like lateral and oblique lateral, occlusal mandibular-maxillary and a three-quarters profile view for esthetic purposes.^14^ Photography can be divided into three broad areas namely: preparation of the patient, background and intraoral sites, preparation of camera.^15^

### Preparation of the Patient

- The patient should be seated comfortably in the chair and explained about the procedure.
- Adjust the height of the chair so that the subjects head is lower than the photographer's head by asking his/her to turn or tilt their head.
- Surgical drape should be changed for every patient to avoid blood stained images.
- If patient helps in retraction, ask him to wear gloves.

### Preparation of Background, Instruments and Intraoral Sites

- Consistent background should be present behind the patient for pre-, mid- and post-treatment photos.
- The area to be photographed should be clean of debris, excess saliva, blood, air bubbles, impression material and cement, glove powder.
- Proper isolation should be done symmetrically with plastic retractors (cheek and occlusal) to obtain unrestricted view.
- Use black spatula to prevent coverage of front teeth by lips with high quality mouth mirrors to aid in better view.
- Mirrors necessary for lateral, palatal and occlusal views should be rhodium coated.
- Dip the mirror in hot water and dry it with cotton or tissue paper alternatively using light stream of air from air syringe to avoid fogging.
- Black backgrounds allow better contrasts without compromising the translucency of teeth and restorations to be displayed.

### Preparation of Camera and Dentist

- Intraoral views should be shooted in landscape mode whereas in extraoral photographs portrait mode is used.
- Use smallest aperture to maximize depth of field, with 1:1 magnification of lens.
- Photograph teeth in correct axial alignment (occlusal plane should be parallel to the horizontal in photograph).
- Keep nose out of palatal view of maxillary incisors.
- Avoid beard hairs.
- Retract tongue with mirror/ask patient to move tongue posterior so as to attain a proper background while photographing teeth.
- Proper cropping should be done to minimize confusion with mirror edges, fingers, unreflected teeth.
- If the photographic conditions are standardized, it is easy to compare then even if they were clicked by different photographers after long time intervals.
- Use manual focus, autofocus is unreliable for oral cavity.
- Eliminate poor quality and over or underexposed images, out of focus and poorly oriented images.

All these parameters should be followed religiously by a dentist for obtaining an excellent photograph. In orthodontics these images provide additional advantage of double-check on errors in band placement and in archwire construction.^16^ Addition of photographic records to quantified postmortem dental charts improvises the accuracy and quality of the report that can be reproduced even if previous data is lost.^17^ Intraoral photographs also proved to be a viable alternative to dental casts for attaining dental arch relationships in cleft lip and palate patients thereby reducing the cost and inconvenience.^18^ Clinical photographs also hold significance in medicolegal cases of personal injury where the approach has to be more professional and adequate coverage is necessary to avoid negligence.^19^Preoperative pictures aids in instrument and site selection so the errors can be avoided in craniofacial surgeries.^20^Measurements can be done on these photos along with shade selection in implants and course of arteries and nerves can be highlighted using Adobe Photoshop software's.^21^

### Guidelines for Photography of Radiographs

Radiographs are only composed of white and black areas. Therefore, an imperfect photograph can be the result of four characteristics, i.e. nonhomogeneous illumination, variable color, flicker and limited brightness.

***Standardization of Viewer Box***

It should consist of 20 watt two fluorescent tubes to illuminate centrally and homogeneously in all corners of the box. If such conditions are not present the tonal range of the camera should be increased, that will help in capturing image with full detail. Cool white tubes with color temperatures around 4000°C helps to balance out the blue-colored radiographic film base to result in neutral color balance. To avoid flicker, correctly circuited tubes should be used and more importantly the camera should be exposed for 1/60th or 1/4 of a second to the film. If this time is not maintained and is less than 1/50th second it gives a yellow cast or banded appearance to the image. On the contrary, longer exposure time may also lead to noise production, i.e. blurring in image. For CT, MRI images blue filters should be used and a sheet of processed, unexposed film should be placed beneath the actual film, so the film base will be blue allowing the filtration of image whereas in less contrast images filters should be avoided. Darker areas on the box could be avoided by the placement of precut props covered with another front face glass. This technique is known as unsharp masking. The camera should be placed atleast 20 cm away from the radiograph. To reduce the disturbances caused by conventional viewer boxes, new LED boxes having properties like high brightness, more than 4000 lux with light source laid out in equal spaced points are available and their uniformity of luminance is above 90%. The light frequency is above 50 kHz which relieves the eye fatigue. It also has an advance clamping film setting which is suitable for films with different thickness. Finally, the ultra thin design (approx. 4.5 cm), power set up inside the viewer and an instrument for desk and wall mounting makes it an effective alternate to age old boxes.^22^ Another quick, feasible method for clicking radiographic photos is by placing the film on a white background of a blank powerpoint presentation as it provides enough illumination and appropriate size.^23^

Teledentistry is a new means of telecommunication and exchange of medical or dental information in remote areas for supervision, diagnosis and public health issues. Digital photographs can be easily transferred from one place to another via networks.^24^ The department of defence, USA has evolved a new program to facilitate total patient care, laboratory investigations and continuing dental education systems and is cost effective.^25^ Such hi-tech programs are being tried in India also to outflow the dental data in rural areas.^26^ But, due to the advent of digital scanners and machinery, dental images can be abused and reformatted to introduce fake restorations, periapical pathoses or full coverage crowns. Dentists, editorial circle and medicolegal insurance companies should be aware of this fact and clinically correlate with the oral findings.^27^ A study conducted on 10 pre- and post-treatment photographs also revealed that such image manipulation through softwares were difficult to detect and correct.^28^ Therefore, a skeptical approach has to be applied in order to expose such frauds in clinical dentistry and research.

## CONCLUSION

The process of dental digital photography is a kind of macro-photography and with the advent of digital cameras; photography has become an easy and accessible way of educating and documenting our patients. Digital images can be easily stored and kept for future use for legal or academic purposes. Therefore, undoubtedly digital cameras should be considered as essential equipment for each dentist and technical as well as photographic training should be inculcated in the curriculum of medical and dental field.

## References

[1] Mladenovic D, Mladenovic L, Mladenovic S (2010). Importance of digital dental photography in the practice of dentistry.. Sci J Faculty Med in Niš.

[2] Terry DA, Snow SR, McLaren EA (2008). Contemporary dental photography: selection and application.. Compend Contin Educ Dent.

[3] Curtin DP (2011). A shortcourses book. The textbook of digital photography..

[4] Goldstein RE (2005). Digital dental photography now?. Contemp Esthet Restor Pract.

[5] Kiran DN, Anupama DK (2010). Digital photography in dentistry.. Indian J Stomatol.

[6] Ho C (2004). Clinical photography: a picture can tell thousand words.. Dent Pract.

[7] Gholston LR (1984). Reliability of an intraoral camera: utility for clinical dentistry and research.. Am J Orthod.

[8] Sachs J (1996). Digital image basics.. Digital Light & Color;.

[9] Clark JR (2004). Digital photography.. J Esthet Restor Dent.

[10] Miot HA, Paixão MP, Paschoal FM (2006). Basics of digital photography in dermatology.. An Bras Dermatol.

[11] Knyaz VA (2012). Image-based 3D reconstruction and analysis for orthodontia. International Archives of the Photogrammetry, Remote Sensing and Spatial Information Sciences.

[12] Nayler JR (2003). Clinical photography: a guide for the clinician.. J Postgrad Med.

[13] Brouwer H, Van Hillegondsberg AJ (1969). Standardized intraoral photography.. JPO J Pract Orthod.

[14] Schaaf H, Malik CY, Howaldt HP, Streckbein P (2009). Evolution of photography in maxillofacial surgery: from analog to 3D photography-An overview.. Clin Cosmet Investig Dent.

[15] Recommended Guidelines for Clinical Photography. Academy of Laser Dentistry's Annual Conference© 2012 Guidelines for Clinical Photography; 2013. Available from:.

[16] Paredes V, Gandia JL, Cibrián R (2006). Digital diagnosis records in orthodontics. An overview.. Med Oral Patol Oral Cir Bucal.

[17] Bernstein ML (1983). The application of photography in forensic dentistry.. Dent Clin North Am.

[18] Liao YF, Huang CS, Lin IF (2009). Intraoral photographs for rating dental arch relationships in unilateral cleft lip and palate.. Cleft Palate Craniofac J.

[19] Bryson D (1994). A guide to medicolegal photography for personal injury claims.. J Audiov Media Med.

[20] Ettorre G, Weber M, Schaaf H, Lowry JC, Mommaerts MY, Peter H (2006). Standards for digital photography in craniomaxillo- facial surgery - Part I: Basic views and guidelines.. J Craniomaxillofacial Surgery.

[21] Dental photography. 2008 [cited 2008 Jan 7];. Available from:.

[22] Whitehouse RW (1999). Use of digital cameras for radiographs: how to get the best pictures.. J R Soc Med.

[23] Vyas MB, Hantodkar NV (2011). Photographing a radiograph: a simple alternative-short communication.. J Indian Academy Oral Med Radiol.

[24] Friedman E (2003). Wi-Fi: What it is and what it isn't.. Telehealth Practice Report.

[25] Philip Girard (2007). Military and VA telemedicine systems for patients with traumatic brain injury.. J Rehabilitation Research and Development.

[26] Reddy KV (2011). Using teledentistry for providing the specialist access to rural Indians.. Indian J Dent Res.

[27] Tsang A, Sweet D, Wood RE (1999). Potential for fraudulent use of digital radiography.. J Am Dent Assoc.

[28] Rao SA, Singh N, Kumar R, Thomas AM (2010). More than meets the eye: digital fraud in dentistry.. J Indian Soc Pedod Prev Dent.

